# Editorial: pH as a signal and secondary messenger in plant cells

**DOI:** 10.3389/fpls.2023.1148689

**Published:** 2023-01-31

**Authors:** Agepati S. Raghavendra, Wenxiu Ye, Toshinori Kinoshita

**Affiliations:** ^1^ Deparment of Plant Sciences, School of Life Sciences, University of Hyderabad, Hyderabad, India; ^2^ Peking University Institute of Advanced Agricultural Sciences, Shandong Laboratory of Advanced Agricultural Sciences at Weifang, Weifang, Shandong, China; ^3^ Institute of Transformative Bio-Molecules, Nagoya University, Nagoya, Japan

**Keywords:** acidification, alkalization, apoplast, cytoplasm, PM H^+^-ATPase, plant cell pH, vacuole, V-ATPase

The pH within a plant cell was traditionally expected to be robust and stable. However, the accruing evidence suggests transient changes in cellular pH could exert short or long-term effects. The changes within a plant cell’s pH were regulated by metabolic processes (Felle, 2001, 2005; Zhou et al., 2021). Alkalinization or acidification was frequently noticed during gravitropism (Felle, 2005), root hair growth (Monshausen et al., 2007), stomatal movement (Gonugunta et al., 2009), pollen tube elongation (Behera et al., 2018), phytohormone signaling (Li et al., 2022), and plant-pathogen interaction (Felle et al., 2004; Song et al., 2022). As a result, cellular pH was often debated as a signal or secondary messenger.

The pH changes may not be uniform across the cell or tissue and may be limited to a particular compartment (apoplast, cytosol, or vacuole). The patterns of pH changes in plant tissues are different, depending on root hairs, pollen tubes, mesophyll, and guard cells. There were counter-arguments about the relevance of cytosolic pH in plant cells during ion flux. The ion flux depended on the membrane potential and not exclusively on apoplastic or cytosolic pH. Further, the pH change depended on buffering. We need to consider both pH values and the buffering strength of the cellular compartment (Oja et al., 1999).

Despite the recurring interest in pH changes, comprehensive reviews were limited. Therefore, we planned a Research Topic to review the patterns of plant cell pH and bring out its possible role as a secondary messenger. The five articles on this Topic emphasized the importance of pH in cellular compartments or organelles and highlighted their relevance to cellular function.

Trinh and Masuda presented a comprehensive picture of the dynamic role of chloroplastic pH, particularly of the lumen. Acidification of the lumen and alkalization of stroma through a photochemical electron transport system ensured the energy supply for carbon assimilation. The changes in pH were light-dependent, typical of photosynthesis. The authors pointed out several protein players, such as KEA3, ion/H^+^ antiporters, and *pgr* proteins, that could sense the pH changes and ensure the smooth functioning of chloroplasts.

Stéger and Palmgren critically evaluated the pH changes occurring during the root hair growth along with those of ROS and Ca^2+^. They observed that while the concerted ROS, Ca^2+^, and pH changes occurred, it was unclear whether the pH changes were the cause or the consequence. Gradients of cytosolic pH, along with ROS and Ca^2+^ drove the growth oscillations in root hairs. The plasma membrane proton ATPase (PM H^+^-ATPase) and plant receptor-like kinases, such as RALF and FERONIA, were involved. The authors pointed out that the PM H^+^-ATPase distribution and resulting pH gradients in pollen tubes could differ from root hairs.

Apoplastic pH in root hairs was considered an integrator of plant signaling during the dynamic responses of roots and crucial in plant responses to hormones and nutrients (Gámez-Arjona et al.). The pH in the apoplast remained acidic under normal growth conditions while becoming alkaline during the restricted growth under abiotic/biotic stress. Modulating apoplastic pH caused changes in cell wall components, ion uptake, electric signaling through slow wave potential, and other elements, such as ROS and Ca^2+^. Again, PM H^+^-ATPase mediated these pH changes.

Kinoshita and Kinoshita crisply dealt with the role and properties of the PM H^+^-ATPase. They emphasized the need for PM H^+^-ATPase in the homeostasis of cellular pH and maintain metabolic processes. The activity of PM H^+^-ATPase depended on protein abundance on the plasma membrane and post-translational modification, particularly threonine phosphorylation at the carboxy terminus. The authors highlighted the light-activation of PM H^+^-ATPase to impact photosynthesis, nutrient uptake, and cytoplasmic streaming (Ding et al., 2021; Kinoshita et al., 2023). It is necessary to further assess and validate PM H^+^-ATPase as a master regulator.

Seidel presented an exhaustive review of the structure and catalytic function of plant vacuolar ATPase (V-ATPase). He identified proteins that can activate or inhibit V-ATPase. These regulatory proteins could provide exciting options to modulate V-ATPase activity in plants. Further research on the intracellular location and assembly mechanism of the V-ATPase complex is necessary to understand and exploit V-ATPase for plant improvement. The presence of several isoforms of V-ATPases made it very difficult to manipulate V-ATPase in plants. We also need to recognize the role and the functions of vacuolar proton translocating pyrophosphatase (V-PPase).

Despite the above authoritative reviews, other pH-related phenomena need further attention. Some of them are alkalization or acidification in different intracellular compartments, modulation by pH of primary/secondary metabolism, and interactions with ion transport, mainly nitrate and calcium. Doubts are often raised about the exact role of cytoplasmic pH if it was a cause or consequence of other signaling events during stomatal opening or closure. A re-examination of time-dependent changes in plant cell pH/ROS/NO/Ca^2+^ would help. Critically designed experiments employing suitable ATPase mutants (*vha/aha*) and cutting-edge innovative sensors to monitor plant cell pH changes (Li et al., 2021; Moreau et al., 2022) can provide answers.

PM H^+^-ATPase and V-ATPase have potential applications in agriculture. Upregulation of PM H^+^-ATPase in rice resulted in enhanced nutrient uptake, stomatal conductance, photosynthesis, and yield (Ding et al., 2021; Zhang et al., 2021). Similarly, overexpression of V-ATPase could restrict stomatal closure, enable osmotic adjustment, and improve plant resilience under abiotic stress (Adem et al., 2017; Wang et al., 2021). It would be exciting to check the effect of overexpressing both PM- and V-ATPase to have the double benefit of improved yield and stress tolerance. We hope that our Research Topic would provide basic information, and provoke the interest of plant biologists in plant cell pH.

## Author contributions

AR wrote the first draft. All authors revised and approved the final manuscript.
